# University of Pennsylvania

**Published:** 1893-12

**Authors:** 


UNIVERSITY OF PENNSYLVANIA.—(VETERINARY DEPARTMENT).

*May —.—Degree V. M. D. (Veterinariæ Medicinæ Doctor).*

| | |
|---|---|
| Connor, John F. - - - | Philadelphia, Pa. |
| Corson, Percy H. - - | New Richland, Minn. |
| Cotton, Charles E. - - - | Prescott, Wis. |
| Earnest, Charles M. - - - | Philadelphia. |
| Fitzpatrick, Dennis B. - - | Philadelphia. |
| Forsyth, George O. - - - | Pemberton, N. J. |
| Greeson, James O. - - | Kokomo, Ind. |
| James, John Alvin - - - | Aberdeen, Md. |
| Jefferis, Joseph R. - - - | Wilmington, Del. |
| Jolly, George O. - - - | Philadelphia. |
| Koenig, August O. - - - | Philadelphia. |
| McCurdy, Frank C., A.B. - - | Tioga, Phila. |
| Magill, Charles E. - - - | Haddonfield, N. J. |
| Patterson, Henry G. - - - | Mifflintown. |
| Paxson, Harry D. - - - | West Chester. |
| Shields, William A. H. - - - | Philadelphia. |
| Smith, Evic A. - - - | Philadelphia. |
| Stuart, James A. - - - | Beverly, N. J. |
| Stuart, George E. H. - - - | Holmesburg. |
| Terry, Edward E. - - - | Trevose. |
| Turner, Henry W. - - - | West Chester. |
| Walls, Alexander C. - - - | St. Paul, Minn. |
| Walter, Harry K. - - - | Point Pleasant. |
| Werntz, William T. S. - - - | Philadelphia. |
| Willganz, Chris. J. - - | Buffalo, N. Y. |
| Young, William, Jr. - - - | Riverton, N. J. |

